# Optimizing noninvasive sampling of a zoonotic bat virus

**DOI:** 10.1002/ece3.7830

**Published:** 2021-08-27

**Authors:** John R. Giles, Alison J. Peel, Konstans Wells, Raina K. Plowright, Hamish McCallum, Olivier Restif

**Affiliations:** ^1^ Department of Epidemiology Johns Hopkins University Bloomberg School of Public Health Baltimore MD USA; ^2^ Environmental Futures Research Institute Griffith University Brisbane Qld Australia; ^3^ Department of Biosciences Swansea University Swansea UK; ^4^ Department of Microbiology and Immunology Montana State University Bozeman MT USA; ^5^ Disease Dynamics Unit Department of Veterinary Medicine University of Cambridge Cambridge UK

**Keywords:** bat virus, sampling bias, under roost sampling, viral prevalence

## Abstract

Outbreaks of infectious viruses resulting from spillover events from bats have brought much attention to bat‐borne zoonoses, which has motivated increased ecological and epidemiological studies on bat populations. Field sampling methods often collect pooled samples of bat excreta from plastic sheets placed under‐roosts. However, positive bias is introduced because multiple individuals may contribute to pooled samples, making studies of viral dynamics difficult. Here, we explore the general issue of bias in spatial sample pooling using Hendra virus in Australian bats as a case study. We assessed the accuracy of different under‐roost sampling designs using generalized additive models and field data from individually captured bats and pooled urine samples. We then used theoretical simulation models of bat density and under‐roost sampling to understand the mechanistic drivers of bias. The most commonly used sampling design estimated viral prevalence 3.2 times higher than individual‐level data, with positive bias 5–7 times higher than other designs due to spatial autocorrelation among sampling sheets and clustering of bats in roosts. Simulation results indicate using a stratified random design to collect 30–40 pooled urine samples from 80 to 100 sheets, each with an area of 0.75–1 m^2^, and would allow estimation of true prevalence with minimum sampling bias and false negatives. These results show that widely used under‐roost sampling techniques are highly sensitive to viral presence, but lack specificity, providing limited information regarding viral dynamics. Improved estimation of true prevalence can be attained with minor changes to existing designs such as reducing sheet size, increasing sheet number, and spreading sheets out within the roost area. Our findings provide insight into how spatial sample pooling is vulnerable to bias for a wide range of systems in disease ecology, where optimal sampling design is influenced by pathogen prevalence, host population density, and patterns of aggregation.

## Graphical abstract

Studies of viruses in bat populations often collect pooled samples of bat excreta from plastic sheets placed under‐roosts. However, multiple individuals may contribute to pooled samples, introducing positive bias, and making studies of viral dynamics difficult. We assess the accuracy of different under‐roost sampling designs and find that improved estimation of true prevalence can be attained with minor changes to existing designs such as reducing sheet size, increasing sheet number, and spreading sheets out within the roost area.

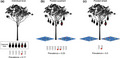

## 1 INTRODUCTION

Recent emergence of bat‐borne viruses has motivated an increase in ecological and epidemiological studies on bat populations at the global scale (Calisher et al., 2006; Halpin et al., 2007; Wang & Cowled, 2015). Initial efforts focused on discovering the reservoir host(s) of these emerging infections (Breman et al., 1999; Chua et al., 2002; Halpin et al., 2000; Jayme et al., 2015; Li et al., 2005; Towner et al., 2009) and identifying other potential viral zoonoses in bats (Anthony et al., 2013; Drexler et al., 2012; Quan et al., 2013; Smith & Wang, 2013). However, less work has been done to describe the dynamics of viruses in bat populations in time and space (Becker et al., 2019). Spatiotemporal sampling is therefore critical to provide insights into the broader ecological context surrounding spillover and to understand the factors that lead to the emergence of bat‐borne viral diseases in humans (Plowright et al., 2019).

A common approach in bat‐borne disease research involves the capture of many individual bats repeatedly over time, where bats are sampled (e.g., serum, urine, feces, saliva) and tested for viral presence using serology or PCR. In the best case scenario, repeated (longitudinal) samples are obtained from individuals, enabling description of dynamics at the individual level. Individual‐level longitudinal data are rare (Becker et al., 2019) and are most often available for high‐fidelity cave‐roosting bats which can be recaptured at the same roosting site (Streicker et al., 2012; Towner et al., 2009). Longitudinal data are very difficult to gather from tree‐roosting megachiroptera, such as the highly mobile nomadic foragers *Pteropus* and *Eidolon* genera (Hayman et al., 2012); therefore, individual‐level sampling in this context is typically done cross‐sectionally over time. Moreover, catching individual canopy roosting bats is logistically challenging and expensive, and therefore, sample sizes are often too small to detect pathogens that circulate at low prevalence. Therefore, much research has supplemented the capture of individual bats with a noninvasive sampling technique that uses plastic sheets to collect pooled samples of bat excreta (e.g., urine and feces) under bat roosts referred to as “under‐roost sampling” (Baker et al., 2012, 2013; Bourgarel et al., 2018; Chua, 2003; Chua et al., 2001, 2002; Edson, Field, McMichael, Jordan, et al., 2015; Field et al., 2011, 2015; Lim et al., 2019; Lima et al., 2013; Marsh et al., 2012; Memish et al., 2013; Mendenhall et al., 2019; Peel et al., 2019; Pritchard et al., 2006; Smith et al., 2011; Valitutto et al., 2020; Wacharapluesadee et al., 2010).

Under‐roost sheet sampling was initially implemented in 1998 to isolate Nipah and Tioman viruses from urine collected from *Pteropus hypomelanus* and *P. vampyrus* in Malaysia (Chua, 2003; Chua et al., 2001, 2002). It has subsequently been widely adopted to study coronaviruses (Bourgarel et al., 2018; Lim et al., 2019; Lima et al., 2013; Memish et al., 2013; Mendenhall et al., 2019; Valitutto et al., 2020) and henipaviruses (Baker et al., 2012, 2013; Edson, Field, McMichael, Jordan, et al., 2015; Field et al., 2011, 2015; Marsh et al., 2012; Peel et al., 2019; Pritchard et al., 2006; Smith et al., 2011; Wacharapluesadee et al., 2010) in bat populations. The most salient complication of under‐roost sampling is that it only provides indirect measures of viral prevalence; that is, viral presence–absence is recorded for a group of bats roosting above a sampling sheet during a certain time period. In this scenario, samples are comprised of urine droplets or fecal particles from an “area” that may be pooled to constitute sufficient volume for an array of molecular assays (i.e., PCR or viral isolation). Although this is a necessary compromise, the clustered nature and fluctuations of bat density within a roost may confound results because an unknown and variable number of individuals contribute to a sample. In lieu of these confounding effects, under‐roost sampling as it is commonly implemented may therefore introduce systematic sampling bias in the form of increased sensitivity to detecting virus.

The increased sensitivity of pooled samples in disease surveillance is well‐known. Sample pooling was first used during World War II to avoid the “expensive and tedious” process of monitoring syphilis in US soldiers (Dorfman, 1943). It has since been used as a cost‐effective method to screen for HIV infection in developing countries (Behets et al., 1990; Litvak et al., 1994), and more recently, it has been employed to increase the efficiency of detecting cases of SARS‐CoV‐2 infection in the ongoing COVID‐19 pandemic (Aragón‐Caqueo et al., 2020; Griesemer et al., 2020; Narayanan et al., 2020). Pooled sample testing is also common in surveillance of agricultural diseases of livestock (Arnold et al., 2005; Christensen & Gardner, 2000), poultry (Arnold et al., 2009; Fereidouni et al., 2012), and aquaculture (Laurin et al., 2019), where a pooled sample is used to determine the presence or absence of a disease within a closed population. The resource efficiency of such pooled sampling techniques stems from heightened sensitivity of quantitative PCR tests within each sample (Muniesa et al., 2014; Muñoz‐Zanzi et al., 2006). In this regard, pooled sampling is well‐suited for disease surveillance because the higher sensitivity is advantageous when pathogen prevalence is very low and access to individuals in the population is hindered. The high sensitivity of pooled samples, however, becomes problematic when used to estimate prevalence (Cowling et al., 1999)—a classic statistical problem resulting from data aggregation, often referred to as the “ecological fallacy” (Robinson, 2009). In the context of under‐roost sampling to estimate prevalence of a bat virus, this sampling conundrum is inherited from the initial application of the under‐roost sampling technique which was to detect and isolate viral agents, not necessarily to study viral dynamics. Still, a few recent studies have employed the technique to describe temporal patterns in viral prevalence (Field et al., 2015; Páez et al., 2017; Peel et al., 2019; Wacharapluesadee et al., 2010); however, the extent to which the data are vulnerable to sampling bias has not been explored.

Here, we contribute the first modeling study to theoretically explore the application of under‐roost sheet sampling to estimating viral prevalence in tree‐roosting bat populations and quantify the potential sampling bias introduced by different sampling regimes. We focus on tree‐roosting pteropodid bats because they are reservoir hosts of henipaviruses which constitute public health risks across Africa, Asia, and Australia; based on their highly mobile population structure, under‐roost sampling techniques are especially useful but also prone to bias due to sample pooling. To show the extent of estimation bias resulting from sample pooling on pathogen prevalence, we fit generalized additive models (GAM) to previously published data of Hendra virus prevalence variation over time in Australian fruit bats at the individual level and two levels of sample pooling. We then developed spatial simulation models of bat density within a tree roost and under‐roost sampling designs and then performed a global sensitivity analysis to assess which aspects of under‐roost sampling impact sampling bias most strongly. Our GAM results show that pooling of urine samples collected with the under‐roost sampling method leads to overestimation of the prevalence of virus. Further, we show that our simulation model elucidates the mechanistic drivers of estimation bias and provides recommendations on how to optimize under‐roost sampling for the surveillance of infectious bat viruses by minimizing bias and maintaining sufficient detection rates.

## 2 METHODS

### 2.1 Estimating viral prevalence from individual and pooled samples

**FIGURE 1:**
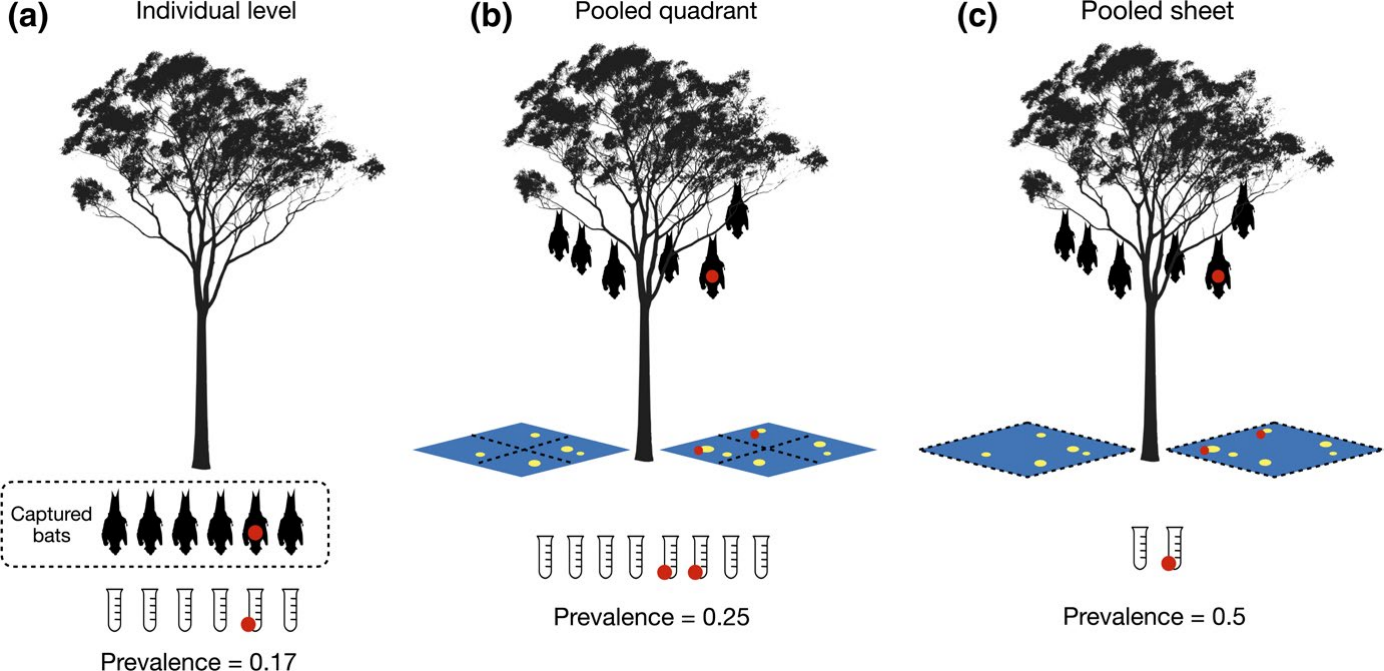
Conceptual drawing of sampling techniques commonly used to estimate viral prevalence at the roost level. Individual‐level sampling is shown in (a) where individual bats are captured and each provides a sample that is used to calculate prevalence. Both (b) and (c) show under‐roost sampling techniques that collect urine droplets from plastic sheets laid beneath roosts. The pooled quadrant technique (b) pools urine droplets that fall within each of the four quadrants of a plastic sheet. The pooled sheet technique (c) pools urine droplets within each plastic sheet. The examples of prevalence calculation show how overestimation of prevalence at the roost level can occur due to multiple bats contributing to a sample. Note that this toy example assumes all bats are captured and the assay used to test samples has perfect sensitivity and specificity

To assess potential sampling bias in estimates of the temporal fluctuations in viral prevalence resulting from pooled samples, we fitted generalized additive models (GAMs; Wood, 2006) to existing “presence–absence” field data of virus detections. From these data, we modelled the probability of viral presence as the response variable and sampling date as predictor variable for different levels of sample aggregation: (a) a broad spectrum of samples from individual bats (i.e., no sample aggregation) and (b) urine samples from multiple roosting bats collected by under‐roost sampling techniques. Due to the intermittent sampling of these data, there are many dates for which data are not available. Fitting GAMs here allows estimation of viral prevalence as smoothed functions and nonlinear response curves over time, enabling comparisons of whether different levels of data pooling would result in different conclusions about the temporal fluctuation in viral prevalence. The field data were collected as part of a Hendra virus study in Australia that collected almost 15,000 urine samples across a 2,300 km latitudinal gradient (Field et al., 2015), methods for data collection have been described in detail elsewhere (Edson, Field, McMichael, Vidgen, et al., 2015; Edson et al., 2019; Field et al., 2011, 2015). The data are comprised of two field survey efforts conducted between June 2013 and June 2014 in Boonah, Queensland, at an urban roost of pteropodid bats (i.e., *Pteropus alecto*, *P. poliocephalus*, and *P. scapulatus*). The first data set measures viral infection and routes of excretion for 1,012 individual black flying foxes (*P. alecto*) captured at the study roost (see Figure 1a). Viral infection was recorded as present if RT‐PCR analyses returned a cycle threshold (Ct) value of <40 for samples taken from any route of excretion (e.g., urine, urogenital, serum, nasal, oral, and rectal; see Edson, Field, McMichael, Vidgen, et al., 2015; Edson et al., 2019). The second data set measured viral prevalence at the roost scale using the under‐roost sheet sampling method where large plastic sheets are divided into quadrants and urine samples are pooled within each sheet quadrant for RT‐PCR testing with the same threshold of Ct <40 for positive samples (see Field et al., 2011, 2015). We used these roost‐scale data to calculate viral prevalence at two levels of sample aggregation: (a) “pooled quadrant” samples which are comprised of pooled urine samples within each sheet quadrant (Figure 1b) and (b) “pooled sheet” samples which are comprised of a combined result for all pooled samples collected from a sheet, that is, across all four quadrants (Figure 1c). Note that the individual bats sampled are not necessarily the same bats that contributed to urine collected via the under‐roost method, though sampling was temporally aligned within 7 days (0–19 95% CI). We fit a GAM with quasi‐binomial error structure and a thin‐plate spline based on date of sampling to the viral presence data collected at the individual, pooled quadrant, and pooled sheet levels (Wood, 2006). We then assessed the smoothed models by calculating the bias in mean estimated viral prevalence of the pooled quadrant and pooled sheet models in comparison with individual‐level model.

### 2.2 Modeling bat population density in a roost

Day roosts of pteropodid bats encompass many trees, with individuals often moving within the roost throughout the day due to mating behaviors or in response to roost disturbance (Kunz & Fenton, 2006), so we modeled bat density within a generic bat roost with a Poisson cluster process of roosting positions and a spatial Gompertz probability density function that reflects movement within a roosting site. Specifically, bat density within roost area *A* (a disk with radius *r*) is constructed in four stages that include the following: (1) placement of roosting trees within the roost area, (2) clustering of individual bats around them, (3) individual‐level movement within a tree, and (4) a separate model of roost‐wide movement. We used a Thomas cluster process to simulate the spatial clustering of bat positions around trees, using the rThomas function from the spatstat package in the R programming language (Baddeley et al., 2015; R Core Team, 2016). Tree locations (parent points) were randomly distributed within *A* subject to a homogeneous intensity κ, given by *n_t_*/*A*, where *n_t_* is the number of occupied trees in the roost. The mean number of bats in each roost tree *µ* is simulated by the cluster point process so that *µ* is stochastic with Poisson distributed error. Individual bat positions are determined according to an isotropic Gaussian kernel centered on each tree with radius *r_t_*. Note that even when parameters κ, *r_t_*, and *µ* are fixed, the number of bats in the roost *N_b_* will still vary upon each simulation because the Poisson point process is stochastic. In simulation scenarios, we chose ranges for parameters of roost structure and bat density based upon unpublished field data and expert observations (see Table 1 for a list of variables used to define each scenario).

**TABLE 1:** Fixed and varied parameter values used in each of the four scenarios

Parameter	Description	Scenario 1	Scenario 2	Scenario 3	Scenario 4	Scenario 5
*n*_sim_	Number of simulations	1,000	1,000	10,000	10,000	1,000
Type	Type of sheet‐based design	QUSR	QUSR	QS	S	Q
*r*	Radius of roost (m)	30	30	Unif(25, 50)	Unif(25, 50)	Unif(25, 50)
*p*	True prevalence	0.1	Unif(0, 1)	Unif(0, 1)	Unif(0, 1)	Beta(0.38, 7.43)^a^
*p_u_*	Probability of urine contribution	0.5	0.5	Unif(0.2, 0.8)	Unif(0.2, 0.8)	Unif(0.2, 0.8)
*S*	Area of sheet^b^ (m^2^)	0.25	0.25	0.25	Unif(0.25, 2)	2.34
*h*	Number sheets placed under‐roost^c^	100	100	100	Unif(25, 150)	10
*d_s_*	Distance between sheets^d^ (m)	2	2	2	Unif(0, 5)	2
*n_s_*	Number of sheets^c^	100	100	100	25–150	–
*n_t_*	Number of occupied roost trees	50	50	Unif(25, 75)	Unif(25, 75)	Unif(25, 75)
*r_t_*	Mean radius occupied roost trees	3	3	Unif(2, 6)	Unif(2, 6)	Unif(2, 6)
*µ*	Mean number individuals per tree	100	100	Unif(25, 150)	Unif(20, 150)	Unif(20, 150)
Shape	Curvature of movement kernel	0.8	0.8	Unif(0.5, 2)	Unif(0.5, 2)	Unif(0.5, 2)
Rate	Movement decay rate at roost edge	1	1	Unif(1, 2)	Unif(1, 2)	Unif(1, 2)

Note

For scenarios 2–5, min and max set the minimum and maximum values of a uniform probability distribution within a random Latin hypercube sampling approach.

Abbreviations: Q, quadrant; R, random; S, stratified; U, uniform.

^a^ Parameters fitted to observe individual‐level Hendra virus prevalence data.

^b^ Small‐sheet designs only. Quadrant‐design fixed at 2.34 m^2^ per sheet quadrant.

^c^ Small‐sheet designs only. Quadrant‐design fixed at 10 sheets with 4 quadrants each.

^d^ Stratified design only.

**FIGURE 2:**
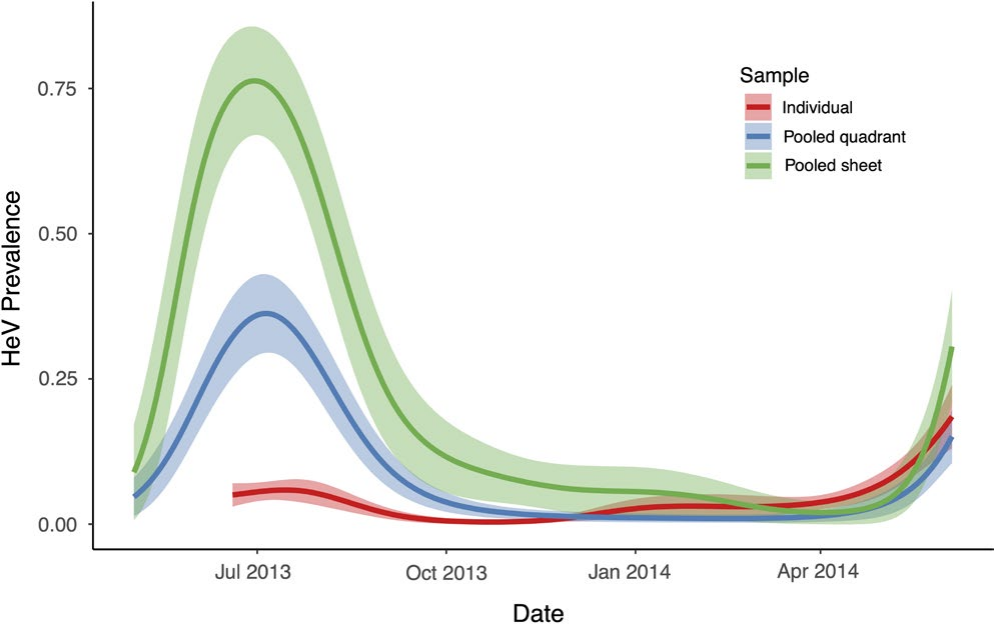
Changes in Hendra virus prevalence over time at a roost in Boonah, Queensland, from May 2013 to June 2014. Solid lines show viral prevalence estimated by generalized additive models (GAMs) fitted to observe field data collected from individually captured bats (red), and under‐roost sampling techniques that aggregate urine samples at the pooled quadrant level (blue) and at the pooled sheet level (green). Note that the GAM for individually captured bats begins in later because the study period for this level of sampling begins in June 2013. See Figure 1 for conceptual drawing of sampling types. Shaded regions indicate the standard error of fitted GAMs. [Correction added on 17 September 2021, after first online publication: Figure 2 caption has been updated in this version.]

Bat movement was modeled at the individual level and roost level (see Figure 2). To model individual‐level movement, we calculated a kernel density estimate for the simulated point process that sums Gaussian kernels with a radius of 0.5 m centered on each bat position. We modeled roost‐wide movement with a spatial Gompertz probability density using the dgompertz function from the flexsurv package (Jackson, 2014). The distribution of the Gompertz is controlled by shape and rate parameters that determine the function's curvature and rate of decay, respectively. We chose ranges for these parameters that make the least assumptions about movement, where values are high for a large area at the roost's center, but decay quickly toward the edges. To make the final kernel density estimate for bat density, we combined models of individual‐ and roost‐level movement and ensured that the function integrated to 1 (Figure 2).

### 2.3 Modeling under‐roost sheet sampling designs

**FIGURE 3:**
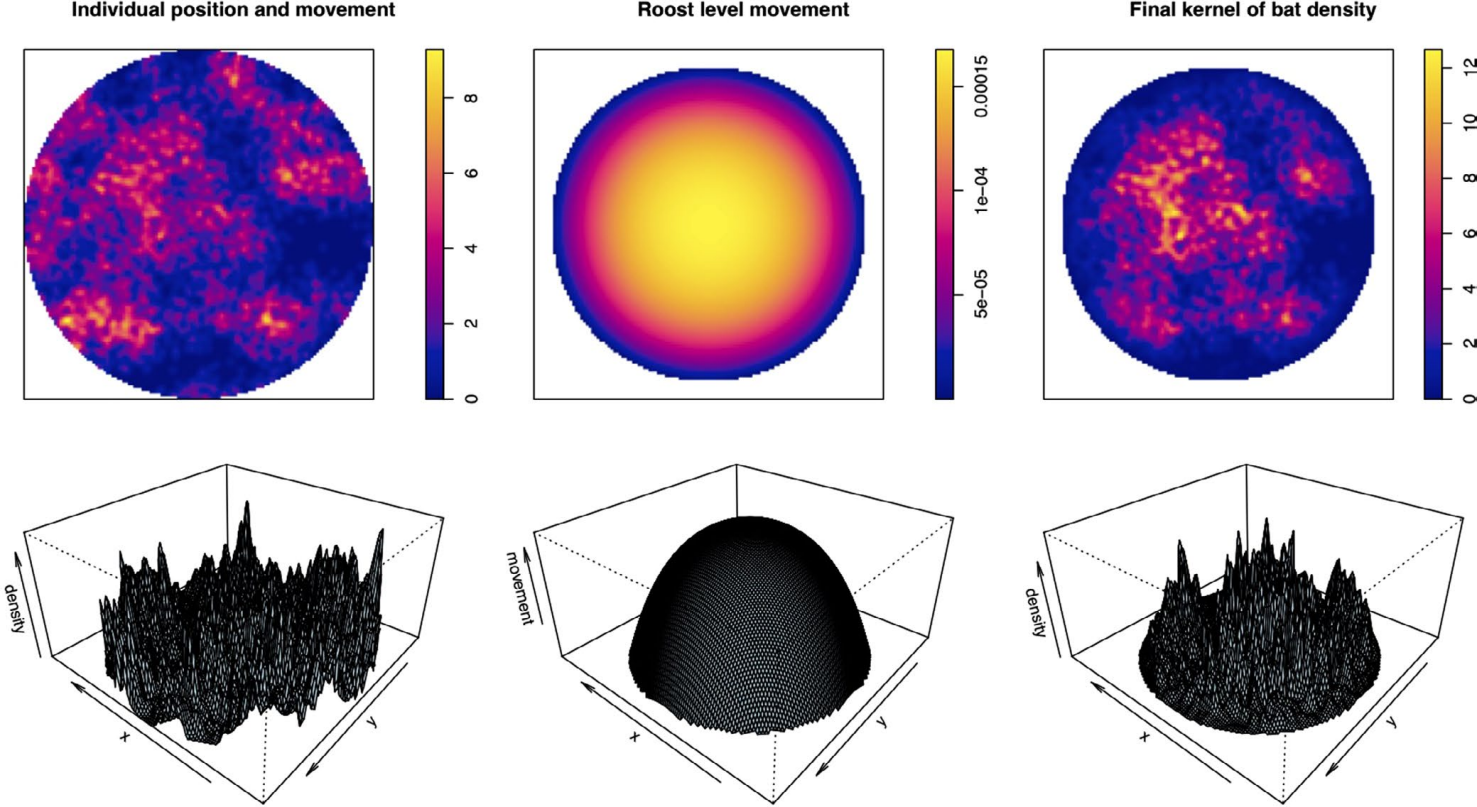
Illustration of one simulation of a kernel density estimation of bat density within a roost. The top row shows pixel images, and the bottom row shows perspective plots of: the density of roosting positions and individual‐level movement around them (left), an isometric Gompertz probability density function centered on the roost to model roost‐level movement (middle), and the final estimated intensity function used to model bat density (right). [Correction added on 17 September 2021, after first online publication: Figure 3 caption has been updated in this version.]

Under‐roost sampling designs typically use large sheets placed under‐roost trees, and urine droplets or fecal particles are pooled into an aggregate sample from the area (or subarea) of each sheet. Most studies provide minimal description of the sheet sampling design; however, Wacharapluesadee et al. (2010), Field et al. (2015), and Edson, Field, McMichael, Jordan, et al. (2015) describe their quadrant‐based sheet design in greater detail (i.e., sheet dimensions, number of sheets, pooling of urine samples). We therefore explored the effect of four different under‐roost sheet sampling designs: quadrant, uniform, stratified, and random (Thompson, 2012) [see Figure 3]. An efficient way to simulate each sampling design within two‐dimensional circular space uses hexagonal tiles, where the size and combination of tiles selected can replicate different sheet‐based sampling designs. We calculated the number of bats roosting and moving above a sampling sheet by using the area of each hexagonal polygon to define the space of integration *S*.

We determined the dimensions for the quadrant‐based design using descriptions of under‐roost sheet sampling of Australian fruit bats found in Field et al. (2015) and Edson, Field, McMichael, Jordan, et al. (2015). Here, 10 large 3.6 × 2.6 m sheets were placed under the roost and divided into 1.8 × 1.3 m quadrants, where urine samples were pooled within each quadrant (allowing up to 4 samples per large sheet). Considering each quadrant to be its own “sheet,” we replicated this sampling design by making a hexagonal grid with each tile area equivalent to a 1.8 × 1.3 m rectangular sheet. Groupings of 4 hexagonal tiles then suffice as a large sheet with 4 quadrants. In each simulation, we generated 10 sheet positions within *A* using a simple sequential inhibition point process with the rSSI function of the spatstat package (Baddeley et al., 2015). To ensure that all sheets retained the same quadrant orientation and that no two sheets were directly adjacent, we generated sheet positions within a disk of *A* − 3m and set the inhibitory radius to 3*s*, where *s* is the hexagonal cell size. The four cell centers nearest each of the 10 simulated point locations comprised the 40 (10 × 4 quadrants) hexagonal tiles for the quadrant‐based design (Figure S2).

To test our hypothesis that a larger number of smaller sheets will estimate roost‐level prevalence more accurately, we generated hexagonal grids with cell size *s* that select *h* number of tiles in a uniform, stratified, or random pattern. Both uniform and random designs are straightforward, but the stratified sampling design was generated using a sequential inhibition point process, where random points are laid down sequentially, retaining only those that are placed further than a specified inhibitory radius *r_s_*. This is similar to a person attempting to lay down sheets randomly with one rule in mind—“Do not place sheets within *r_s_* distance of each other.” We simulated sheet sampling designs with the sheetsamp function in the R code provided in Supporting Information. Figure 3 displays an example of a simulation which has generated the previously implemented large‐sheet quadrant design and three additional “small‐sheet” designs that use a larger number of smaller (1 × 1 m) more dispersed sheets.

### 2.4 Calculating estimated prevalence

Given a roost area *A*, the polygons produced by the sheetsamp function (described above) generate the sheet sampling area *S*, so that S⊂A, and $S_{h}=\left\{S_{1},S_{2},\ldots ,S_{H}\right\}$, where *H* is the total number of sampling sheets. We derived bat density from a simulated Poisson cluster point process and then estimated its intensity function *λ*(*x*) for area *A*. This method uses kernel density as an unbiased estimator of *λ*(*x*), which includes clustering of bats around trees, individual‐level movement within the tree canopy, and roost‐level movement to render $\tilde{\lambda }\left(x\right)$. The expected number of bats roosting and moving above a specific sheet *S_h_* placed at position $\left(x_{h},y_{h}\right)$ is the integral of the estimated intensity function $\tilde{\lambda }\left(x\right)$ over the sheet area multiplied by the number of bats *N_b_* generated by the stochastic point process.

(1) $$E\left[N\left(S_{h}\right)\right]=\int_{S_{h}}N_{b}\tilde{\lambda }\left(x\right)dx$$

Bats in the upper strata of the canopy are less likely to contribute urine to the sheet below because of obstruction by individuals below or factors in the environment (e.g., wind, tree branches). Therefore, a urine sample is collected from each of the sheets *S* according to a probability of urine contribution and collection *p_u_*, with variation given by *N*(*p_u_*, *σ* ^2^). The number of individuals contributing to each pooled sample *C_b_* is calculated as

(2) $$C_{b}=\int_{S_{h}}p_{u}N_{b}\tilde{\lambda }\left(x\right)dx,$$

where *C_b_* is a vector of length *H*, containing the number of contributing bats per sheet.

Assuming heterogeneous prevalence within the roost, the number of infected bats *D_b_* in the sample is the sum of *C_b_* independent Bernoulli trials with success probability equal to the true prevalence *p*.

(3) $$D_{b}=\sum_{i=1}^{C_{b}}{\left[\text{Bin(}1,p)\right]}_{i}$$

Given the number of infected bats *D_b_* and the probability of urine collection *p_u_*, we can calculate the probability of obtaining a negative sheet as ${\left(1‐p_{u}\right)}^{D_{b}}$. Assuming that urine contribution from one infected bat is sufficient to make a sheet sample positive, the infection status of all sheets is a binary vector *I_h_* indicating the positivity for the *H* sheets of *S*.

(4) $$I_{h}=\left\{\begin{array}{cc} 0, & \text{if}\;D_{b}=0\\ 1, & \text{if}\;D_{b}\geq 1 \end{array}\right.$$

To calculate estimated sheet‐level prevalence $\hat{p}$, the number of positive sheets $\sum_{h=1}^{H}I_{h}$ is divided by the number of urine samples collected at the roost *n_s_*, which is the sum of a binary vector indicating that the urine of one or more individuals was contributed and collected for all of the *H* sheets of *S*.

(5) $$\hat{p}=\frac{\sum_{h=1}^{H}I_{h}}{n_{s}},$$

where

(6) $$n_{s}=\sum_{h=1}^{H}{\left[C_{b}\geq 1\right]}_{h}.$$

### 2.5 Simulation scenarios of bat population density and under‐roost sampling

Each simulated iteration generates an estimated intensity function for bat density and then performs under‐roost sampling using each of the four sampling designs. Therefore, each sampling design is tested using the same set of bat density functions, facilitating comparison. Parameters for sheet size *s* and number of sheets *H* were fixed for the quadrant‐based design to replicate the previously implemented field methods described above. Parameters controlling sampling dimensions for the three small‐sheet designs were either fixed or varied over a range of plausible values depending on the question the simulations were meant to address—see Table 1 for a list of parameter values used in each scenario. For each iteration, we calculated estimated prevalence $\hat{p}$ of each under‐roost sheet sampling technique and its bias as an estimator of true prevalence $\left(\hat{p}‐p\right)$. We also calculated additional metrics such as the probability of obtaining a negative sheet ${\left(1‐p_{u}\right)}^{D_{b}}$, the occurrence of a false negative (${\hat{p}}_{i}=0|p_{i}>0$), Moran's I among sheets (Getis, 1973), and the Clark‐Evans R clustering coefficient for individual bat roosting positions (Clark & Evans, 1954).

In the scenarios 1 and 2, we explored local sensitivity between estimated prevalence and some possible confounders and sources of bias, with values of other parameters fixed. To perform a simple comparison between the four under‐roost sheet sampling methods, we fixed all values of bat density and movement to simulate a roost with a 30 m radius and a mean number of 5,000 individuals (see scenario 1 in Table 1). We performed 1,000 simulations with true prevalence *p* set at a plausible value of 0.1 according to mean prevalence estimated for roosts near the QLD‐NSW border in Field et al. (2015). Estimated prevalence values were plotted, along with the probability of obtaining a negative sheet for each sampling design. To explore estimation bias over all values of true prevalence, we kept parameter values the same as scenario 1, but we allowed true prevalence to vary from 0 to 1, and then plotted true versus estimated prevalence along with mean estimation bias (scenario 2 in Table 1).

In scenarios 3 and 4, we performed a large number of simulations (*n*_sims_ = 10,000) and allowed parameter values for each simulation to vary using Latin hypercube sampling. We then analyzed the output using boosted regression trees (BRTs; De’ath, 2007; Elith et al., 2008) as a global sensitivity analysis (described in Prowse et al. (2016)) to identify the main sources of estimation bias and determine the optimal application of under‐roost sheet sampling. Here, to link simulation inputs (varied parameters) with simulation outputs (we used estimation bias and false‐negative rate as responses). Parameter values were randomly sampled using the randomLHS function in the lhs package (Carnell, 2016), and BRTs were fitted using the gbm.step function and the gbm and dismo packages (Hijmans et al., 2016; Ridgeway, 2016). BRTs were fitted with appropriate error structure (Gaussian or Binomial) and meta‐parameters set to ensure that the number of fitted trees exceeded 1,000, following Elith et al. (2008), with tree complexity, learning rate, bagging fraction, and number of cross‐validation folds set to: 4, 0.005, 0.7, and 10, respectively. BRTs act as an effective emulator here because they fit complex nonlinear relationships with up to third‐order interactions (tree complexity = 4) among model parameters. Relative variable influence and individual response curves for each variable further allow general description of how sensitive estimation bias is to each parameter.

In scenario 3, we compare the quadrant‐based design with the stratified design while accounting for the variability in all other parameters to determine the main drivers causing differences in estimation bias. We chose to use only the stratified design as a candidate small‐sheet design because the first two simulations suggested that the three small‐sheet designs produce similar results, and the stratified design is most plausibly replicated in the field. Based on preliminary models, it appeared that a small‐sheet sampling design which used ~100 sheets with an area of ≤1 × 1m^2^ could attain low estimation bias. So, we fixed the parameters controlling sheet dimensions accordingly to facilitate comparison between the quadrant and stratified methods (see simulation 3 in Table 1).

To explore the optimal application of the stratified sampling design, we performed a global sensitivity analysis using only the stratified sampling design in scenario 4. All parameters were varied as in scenario 3; however, sheet area *s*, number of sheets *H*, and distance between sheets (*d_s_*; previously fixed at 2 m) were also varied over intervals of interest (scenario 4 in Table 1). We used a Latin hypercube to sample the parameter space and then fitted two BRT models using the variables that control the sheet sampling design as predictors (i.e., sheet area, number of sheets, distance between sheets, and number of samples): the first model we fitted with Gaussian error and estimation bias as the response and the second with Binomial error and a binary response indicating occurrence of a false‐negative prediction for viral presence.

We validated the theoretical model of bat density and under‐roost sheet sampling in scenario 5, where we simulated values of true prevalence that were based on the distribution of observed values of Hendra virus prevalence in the individual‐level field data. We simulated the individual‐level data by fitting a Beta distribution to observed values of prevalence using maximum‐likelihood estimation and then used this distribution in the Latin hypercube sample of the parameter space (see scenario 5 in Table 1). We then used the quadrant‐based sheet sampling design to match the under‐roost sampling techniques that produced the pooled quadrant level and pooled sheet‐level data (see Field et al., 2011, 2015). To assess how well this scenario simulates the observed field data, we then calculated the mean bias of the pooled quadrant and pooled sheet sampling methods for all simulations and compared them with the observed bias in the field data.

## 3 RESULTS

**FIGURE 4:**
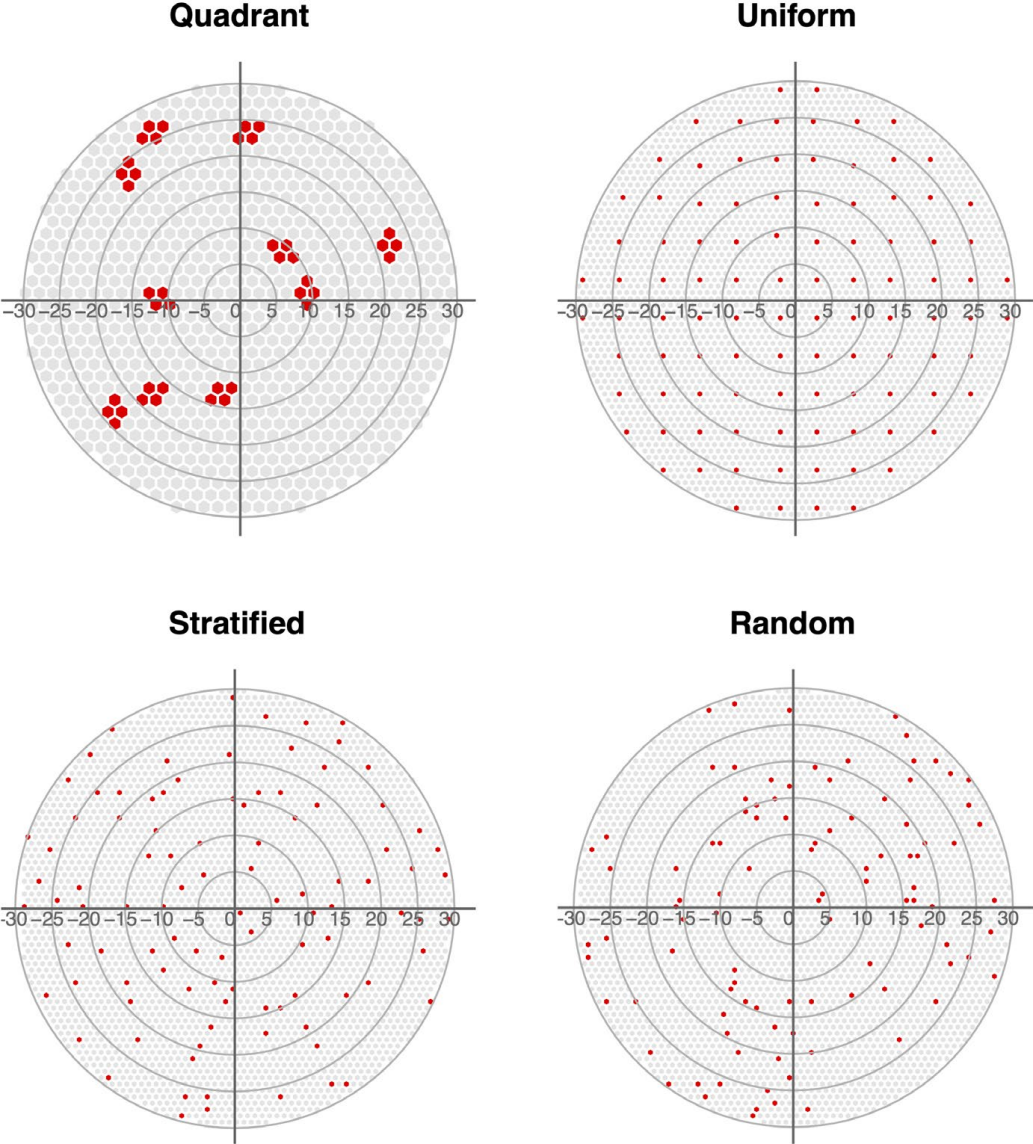
Examples of one simulation of each of the four under‐roost sheet sampling designs explored in this study generated for a roost with a 30 m radius. The quadrant design (top left), which follows methods found in previously published studies (Edson, Field, McMichael, Jordan, et al., 2015; Field et al., 2011, 2015), is comprised of 10 3.6 × 2.6 m sheets, each divided into 1.8 × 1.6 m quadrants for pooling urine samples. The other three designs (uniform, stratified, and random) are all “small‐sheet” designs that reduce sheet area, increase sheet number, and disperse sheets about the roost area. The small‐sheet designs plotted above each contain 100 one‐m2 sheets. The stratified design is generated using a sequential inhibition process with and inhibitory radius of 2 m. [Correction added on 17 September 2021, after first online publication: Figure 4 caption has been updated in this version.]

Fitting of GAMs to field data provided smoothed estimates of Hendra virus prevalence in individual bats and in pooled urine samples collected using under‐roost sampling methods at the Boonah, Queensland, study roost from May 2013 to June 2014 (Figure 4 and Figure S1). The data capture cycle dynamics at this roost with a clear peak in prevalence from June to August 2013 in which the GAM using data from individually captured bats (*P*. *alecto*) estimated to be ≈ 0.1, where GAMs fitted to data collected using under‐roost sampling methods fitted values of prevalence that were considerably higher (pooled quadrant level ≈ 0.4 and pooled sheet level ≈ 0.75; see Figure 4). Over the time span of field sampling, we found the mean bias of the under‐roost method (measured as the difference in the mean estimated viral prevalence of the GAM fitted to individual‐level data compared with the models fitted to under‐roost data) to be 0.07 (−0.04 to 0.35 95% CI) for the pooled quadrant level and 0.21 (−0.02 to 0.71 95% CI) for the pooled sheet level. The resulting magnitude of the bias in prevalence estimates was on average 3.2 times higher (0.31–6.5 95% CI) when using the pooled quadrant‐level data and 8.5 times higher (0.47–23.2 95% CI) for the pooled sheet‐level data (see Figure 1 for sampling techniques and Figure 4 for fitted models). Unsurprisingly, these models indicate that under‐roost sampling methods that use the quadrant‐based design to sample tree‐roosting fruit bats are indeed prone to overestimation of viral prevalence. Further, when we used the Beta distribution—fitted to observed values of viral prevalence from the individual‐level data—as values of true prevalence in under‐roost sampling simulations (see scenario 5 in Table 1), we obtained similar estimates of sampling bias for the quadrant‐based sheet design (Table S1). This simulation scenario estimated the mean bias in prevalence to be 0.06 (−0.06 to 0.38 95% CI) at the pooled quadrant level and 0.21 (−0.06 to 0.73 95% CI) at the pooled sheet level. This amount of estimation bias produced estimates of viral prevalence that were on average 2.5 times higher (0–12.1 95% CI) for the pooled quadrant level and 6.9 times higher (0–39.4 95% CI) for the pooled sheet level compared with simulated values of true prevalence. While the confidence intervals in the simulated data are larger than those observed in the field data, the values of mean bias are closely comparable which provides validation for using the theoretical models to assess optimal under‐roost sampling designs.

When we compared the quadrant‐based sheet design to the small‐sheet designs with fixed model parameters (scenario 1 in Table 1), we found that at a low value of true prevalence (0.1), the quadrant design exhibited strong positive bias and all three small‐sheet designs produced similar estimates close to the fixed value of true prevalence (see top row of Figure S3). The differences in estimated values can be partially attributed to the increased number of bats that roost and move above the larger sheets, which decrease the probability of obtaining a negative sheet (see bottom row of Figure S3). Local sensitivity analysis revealed that, at a low value of true prevalence, prevalence estimation for the quadrant‐based design is sensitive to spatial autocorrelation among sheets (Moran's I) and clustering of bat roosting positions (Clark‐Evans R; Figures S4 and S5). However, the small‐sheet designs are sensitive to the number of bats in the roost (*N_b_*; Figure S6). This indicates that, at low values of true prevalence, the quadrant‐based method remains sensitive to viral presence regardless of the roost population size, but will tend to overestimate viral prevalence due to the spatial clustering of individuals common to most tree‐roosting bats. Conversely, small‐sheet methods appear less affected by clustering and spatial autocorrelation among sheets, but they are likely to be less sensitive to viral presence at low population sizes.

**FIGURE 5:**
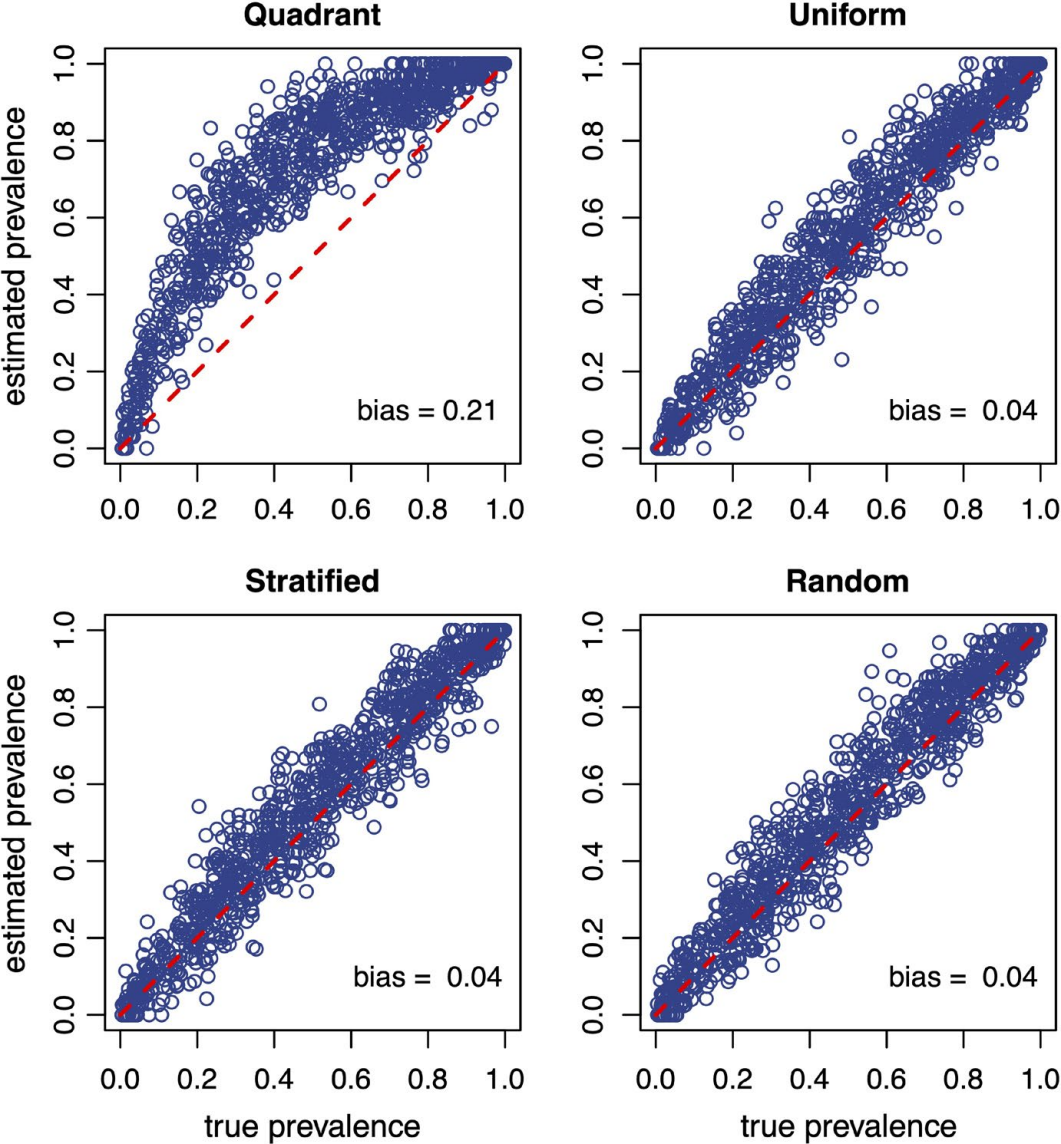
Results of 1,000 simulations performed over all possible values of true prevalence for four different under‐roost sheet sampling designs (see scenario 2 in Table 1). The dashed red line indicates $\hat{p}=p$, and mean estimation bias for all simulations is printed in the lower right corner of each plot

In scenario 2, where we allowed true prevalence to vary between 0 and 1 (Table 1), we found that the quadrant design had 5–7 times the positive bias as the small‐sheet designs. The mean estimation bias was 0.21 for the quadrant design, and 0.04, 0.03, and 0.04 for the uniform, stratified, and random designs, respectively (Figure 5). This suggests that, for a roost size of 3,000–8,000 bats, the estimation bias will consistently be greater for the quadrant design, especially for intermediate values of prevalence. Additionally, the similarity among the uniform, stratified, and random designs indicates that the exact spatial pattern of the small‐sheet method is not important—estimation bias is improved by reducing sheet size, increasing the number of sheets, and spreading sheets out within the roost area. Using these sampling strategies to reduce estimation bias allows under‐roost sampling techniques to more effectively emulate individual‐level sampling.

**FIGURE 6:**
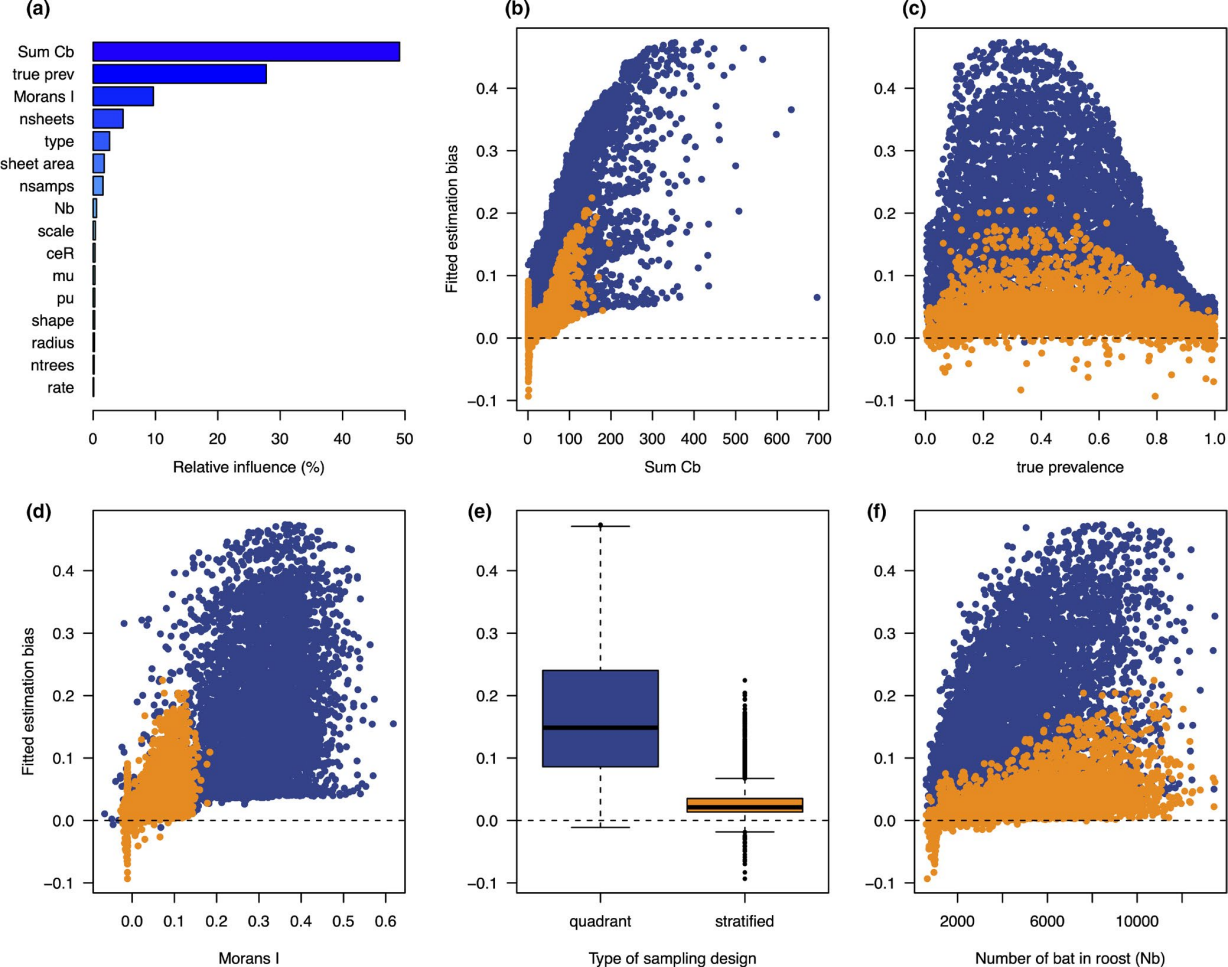
Results of the global sensitivity analysis performed in scenario 3, where the quadrant (blue points) and stratified (orange points) designs are compared to determine what drives differences in estimation bias between the two designs. Table 1 shows the parameters used in the simulation. The barplot (a) shows the relative influence of each parameter determined by a boosted regression tree emulator. Plots e and f show the value of estimation bias fitted by the emulator as a function of five influential parameters (blue: quadrant, orange: stratified sampling design)

Scenario 3 showed significant differences in estimation bias between quadrant and stratified designs, even when we allowed all parameters to vary (Figure 6e). Summary of simulation output with the BRT emulator showed higher bias for the quadrant design, which is most strongly influenced by the total number of individual bats sampled across all sheets ($\sum C_{b}$; Figure 6a,b). This suggests that the larger sheet area in the quadrant design allows pooling of urine samples from more individuals, making the prevalence estimates more sensitive to increases in population size. Further, a quadrant‐based design allows up to four “independent” pooled samples to be adjacent each other, effectively inflating the number of positive sheets, illustrated by higher estimated prevalence associated with high values of Moran's I in Figure 6d. In general, both sampling designs are positively influenced by intermediate values of true prevalence, number of bats in the roost (leading to a greater number of total bats contributing to each sample), and spatial autocorrelation among sheets. However, the influence of these factors is diminished in the stratified design, as shown by the orange points in Figure 6b–f.

**FIGURE 7:**
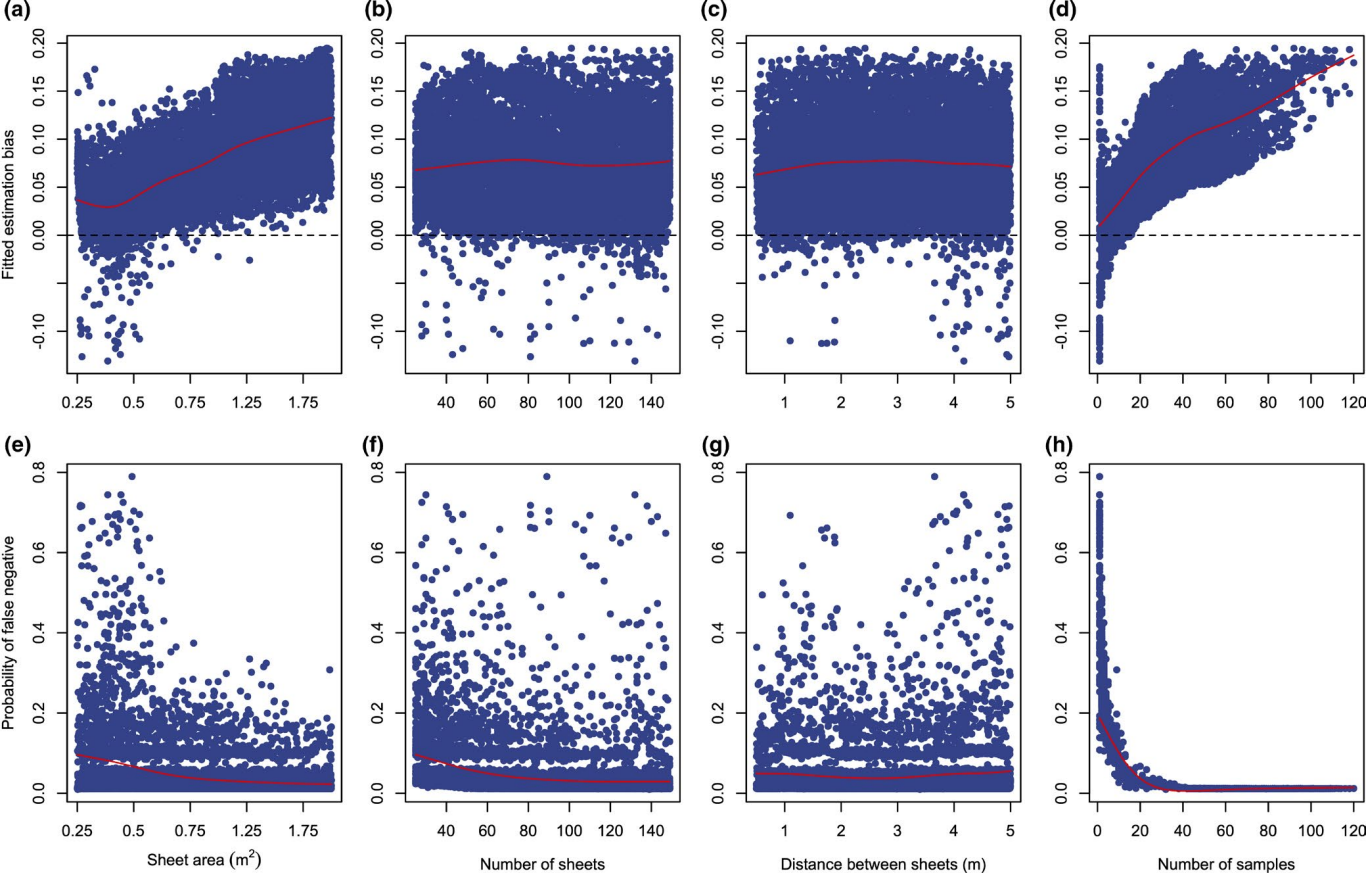
Global sensitivity analysis of scenario 4, where the influence of sheet dimension parameters is explored to determine optimal application of the stratified sheet sampling design. The plots display results from two boosted regression tree emulators: one for estimation bias (top row) and the other for the probability of false negatives (bottom row). Each response is plotted against sheet dimension parameters (from left to right): sheet area *s*, number of sheets *h*, minimum distance between sheets *d_s_*, and number of samples collected *n_s_*. The red lines indicate the trend of the points given by smooth spline regression (sreg function in the fields R package; Nychka et al. (2015))

When we further explored the influence of sheet dimensions for the stratified design (scenario 4 in Table 1), we found that sheet area *s* and number of samples collected *n_s_* influenced estimation bias and probability of false negatives the most, and the number of sheets *H* and distance between sheets *d_s_* had less influence (Figure 7). Specifically, estimation bias increases for sheet area >0.5 m^2^, but the probability of false negatives increases for sheet area <0.75 m^2^. Suggesting that sheet areas in the range of 0.5–1 m^2^ would provide a balance of the two sources of sampling bias (Figure 7a,e). The number of sheets had no influence on estimation bias; however, sampling designs with less than 80 sheets had higher probability of false negatives (i.e., probability of not detecting the virus when it is in fact present; Figure 7b,f). Minimum distance between sheets did not have a significant effect on either source of sampling bias; however, distances between 2 and 3 m fitted the lowest maximum probability of false negatives (Figure 7b,f). The number of samples collected *n_s_* exhibited the largest influence among sheet dimension parameters. Estimation bias increased with a larger number of collected samples, with the possibility for underestimation when under ≈30 samples were obtained (Figure 7d), and the probability of false negatives increased below 30–40 samples (Figure 7h). In general, these results indicate that collecting 30–40 pooled urine samples with a stratified sheet sampling design that uses 80–100 sheets, each with an area of 0.5–1 m^2^, that are separated by a minimum distance of 2–3 m, would provide optimal application of the under‐roost sampling technique that minimizes error introduced by estimation bias and false negatives. Further, we calculated the proportion of simulations matching the parameters stated above and found that, given a roost population size >5,000, 89% of simulations had at least 30 sheets that collected a urine sample, and 64% collected at least 40 samples (Figure S7).

## 4 DISCUSSION

Under‐roost sampling of bat viruses has been employed previously in Africa, Asia, and Australia; however, little attention has been given to the effects of sampling bias or optimization of sampling designs. We used data from field studies of Hendra virus in Australia, which have been extensively studied at both the individual and roost scales to describe temporal and spatial dynamics of viral transmission in bat populations (Edson, Field, McMichael, Vidgen, et al., 2015; Edson et al., 2019; Field et al., 2011, 2015; Smith et al., 2011) and ecological drivers of excretion into the environment (Giles et al., 2018; Páez et al., 2017). We combined these data to compare viral prevalence estimated using individual‐level data to that estimated at two levels of sample pooling and found that systematic pooling of urine samples can lead to overestimation of viral prevalence (Figure 4 and Figure S1). We also show that theoretical models of bat density and under‐roost sampling can replicate patterns of estimation bias observed in field data, allowing us to use simulations to optimize under‐roost sampling designs. Previous work has elucidated factors contributing to sampling bias of zoonotic diseases on larger temporal and spatial scales and noted the importance of targeted sampling designs (Plowright et al., 2019), but to our knowledge, this is the first study to use data and models to investigate the impact of sampling bias on the estimation of viral prevalence in bat populations at the roost scale.

The simulation scenarios we developed provide insight into the mechanistic drivers of estimation bias associated with under‐roost sampling in a theoretical population of tree‐roosting bats. First, sampling designs which use large sheets (larger than ~1 m^2^) and/or sheet quadrants to pool urine samples are sensitive to viral presence, but they potentially overestimate viral prevalence with a bias up to 7 times greater than a design with a greater number of smaller sampling sheets (Figure 5). Second, estimation bias is affected by the number of individuals allowed to contribute to a pooled sample and spatial autocorrelation among sampling sheets; however, these sources of bias can be reduced by adjusting the sheet sampling design (Figure 6). And third, assuming a roost population size of over 5,000 bats, estimation bias can be sufficiently reduced by collecting 30–40 pooled urine samples using a stratified sheet sampling design that uses 80–100 sheets, each with an area of 0.75–1 m^2^, that are separated by 1–3 m (Figure 7 and Figure S7). While field conditions may impact the total number of sheets that can be placed under a roost, our results indicate that the large number of sheets increases the likelihood that a urine sample is contributed to a sheet despite the smaller per sheet area. These insights from simulation models enable well‐informed hypotheses about the optimal sheet design for under‐roost sampling, which can help to refine the application of under‐roost sampling in the surveillance of infectious viruses in wild bat populations.

Our recommendations to optimize under‐roost sampling differ from those previously implemented in the field in that they reduce the size of sheet area, increase the number of sheets, and disperse them about the roost area. In relation to the best‐described methods in the literature, this is roughly equivalent to halving the size of sheet quadrants in Field et al. (2015) and Edson, Field, McMichael, Jordan, et al. (2015) to make 80 0.9 × 0.8 m sheets, and then separating each of them by 1–3 m. Or relative to Wacharapluesadee et al. (2010), the sheets could remain 1.5 × 1.5 m (or be reduced to 1 × 1 m), but the total number of sheets could be increased by 3–4 times. McMichael et al. (2017) explored a modified under‐roost sampling technique where they tested individual droplets to minimize the risk of multiple individuals contributing to a sample, but this requires low bat density and returns small sample volumes, which limits larger‐scale application. Therefore, “optimal” application of an under‐roost sampling design is still inherently limited to pooled sheet‐level estimates of prevalence. We also acknowledge that local topography around a roost can make implementation challenging. Local factors at the roosting site (e.g., physical obstructions, understory vegetation, slope) must be considered when applying sampling designs in the field. This highlights the difficulty in entirely removing positive bias associated with under‐roost sampling of bat viruses; however, it can be mitigated with a sampling strategy that reduces the area of urine pooling and limits spatial autocorrelation among sheets.

Overall, our results indicate that under‐roost sampling designs as they have been applied in the past are poorly suited to studying viral dynamics because of positive sampling bias. For example, Páez et al. (2017) analyzed data from an under‐roost sampling study (Field et al., 2015) and noted that a large amount of variation in viral prevalence was explained by differences in sampling sheets, indicating that population structure within roosts or sampling bias may have introduced additional variation in estimated prevalence. In light of the results from our simulation models, pooling urine samples drawn from large‐sheet areas effectively inflates the number of Bernoulli trials in each Binomial sample, which then increases the sensitivity of detection at the roost level. This may be observed as overestimation when the pooled samples are subsequently used to calculate roost‐level prevalence in field studies. Although we focus on roost‐level sensitivity here, we note that sample pooling could also impact assay sensitivity within a sample through the dilution (i.e., multiple species contributing to a sample) or concentration (i.e., through partial evaporation) of urine or fecal matter on plastic sheets. Collecting pooled samples from a smaller sheet area may therefore reduce the number of bats contributing to a sample, which may require practical consideration of sample volume for required assays. Therefore, these small‐sheet sampling designs have the potential to reduce overestimation, with the caveat that smaller sheets are less likely to collect adequate sample volumes, necessitating a larger number of sheets placed under the roost.

While the data and models presented here focus on testing pooled urine samples, positive bias associated with under‐roost sampling designs also applies to viral pathogens found in bat fecal samples (Ge et al., 2012). Given that bats have been implicated as the probable natural hosts of coronaviruses from which SARS‐CoV‐2 emerged to cause the global COVID‐19 pandemic (Zhou et al., 2020), there has been a call for increased surveillance of novel coronaviruses in wild bat populations (Wacharapluesadee et al., 2021). In scenarios where surveillance of coronaviruses (or other viral pathogens) aims to estimate viral prevalence using excreta collected with under‐roost sampling designs, the modeling techniques we have employed here can be applied to optimize the sampling strategy. Optimization of noninvasive sampling in this context will be an important tool to balance surveillance efforts required for public health with conservation of wild bat populations.

We have shown that sheet design in under‐roost sampling can have a significant impact on both the estimation of viral prevalence and the false‐negative rate when determining viral presence. The sampling design employed, therefore, depends on the aim of the study, because viral discovery and studies on dynamics require different approaches. Research focusing on viral discovery requires field methods that reduce the probability of a false negative regarding viral presence (sensitivity). Studies on dynamics must estimate prevalence with low bias, requiring samples that are accurately classified as present and absent (specificity). Therefore, if a study includes multiple aims, an efficient adaptation of a small‐sheet design includes pooling urine over multiple spatial scales, with samples pooled over a large area to test for viral presence with high sensitivity *and* samples pooled over a small area for estimating prevalence with high specificity. This type of multistage approach is analogous to “herd‐level” testing where a pooled sample is used to determine the presence or absence of a disease, if a pooled sample is found positive, individual‐level samples are then used to identify infected individuals or calculate prevalence more accurately (Martin et al., 1992). Multistage sample pooling may be especially useful for other disease systems where individual capture of free‐ranging host species is not practical, such as aquatic animals (Laurin et al., 2019; Sabino‐Pinto et al., 2019), poultry (Arnold et al., 2009; Fereidouni et al., 2012), livestock (Arnold et al., 2005; Christensen & Gardner, 2000), and wildlife (Walton et al., 2016). Given the challenges associated with under‐roost sampling, our simulation models and recommendations for a small‐sheet sampling design provide specific changes to existing methods that facilitate further adaptation of sampling designs in a model‐guided fieldwork approach (Restif et al., 2012). If applied in a manner suited for study aims, it can achieve longitudinal sampling of a bat population at the roost scale that is both cost effective and reduces exposure to infectious viruses.

## CONFLICT OF INTEREST

None declared.

## AUTHOR CONTRIBUTION

**John R. Giles:** Conceptualization (lead); Formal analysis (lead); Methodology (lead); Visualization (lead); Writing‐original draft (lead); and Writing—review & editing (lead). **Alison J. Peel:** Conceptualization (equal); Data curation (equal); Project administration (equal); Supervision (equal); Validation (equal); and Writing—review & editing (equal). **Konstans Wells:** Conceptualization (equal); Investigation (equal); Methodology (equal); Validation (equal); and Writing—review & editing (equal). **Raina K. Plowright:** Conceptualization (equal); Data curation (equal); Funding acquisition (equal); Project administration (equal); and Writing—review & editing (equal). **Hamish McCallum:** Conceptualization (equal); Data curation (equal); Methodology (equal); Resources (equal); Validation (equal); and Writing—review & editing (equal). **Olivier Restif:** Conceptualization (equal); Funding acquisition (equal); Investigation (equal); Project administration (equal); Resources (equal); Supervision (equal); Validation (equal); and Writing—review & editing (equal).

## Supporting information

Fig S1

Fig S2

Fig S3

Fig S4

Fig S5

Fig S6

Fig S7

Table S1

## Data Availability

Code and data to reproduce models and simulations in this paper can be found at https://github.com/gilesjohnr/batsamphttps://github.com/gilesjohnr/batsamp (Giles et al., 2021).
