# Supplementary figures and images for: Optimizing noninvasive sampling of a zoonotic bat virus
Source: Ecol Evol. 2021 Aug 27;11(18):12307–21. doi: 10.1002/ece3.7830 (PMC8462156; doi:10.1002/ece3.7830)

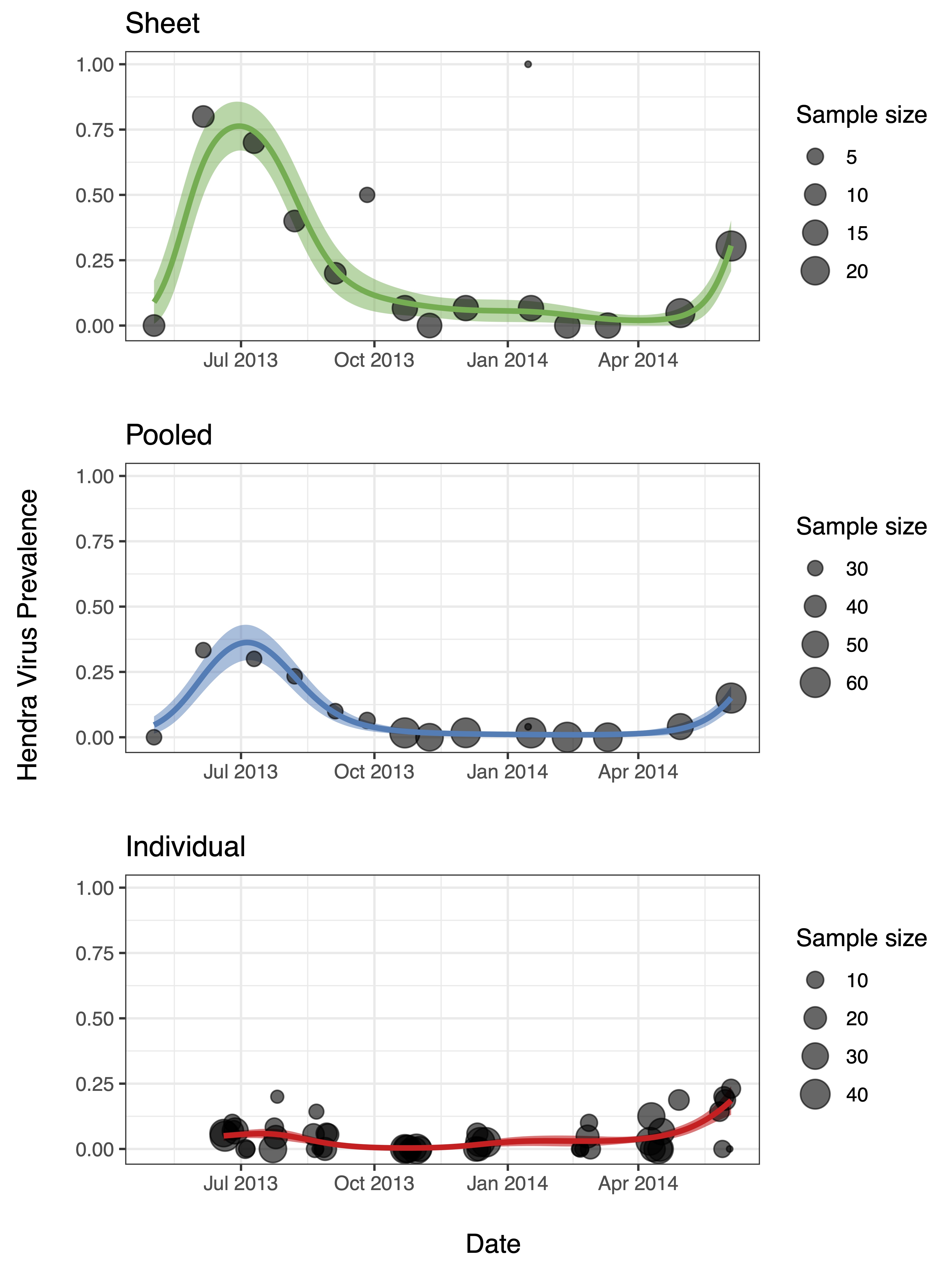

Supplement: Supplementary file 1 — Fig S1 [file ECE3-11-12307-s005.jpg]

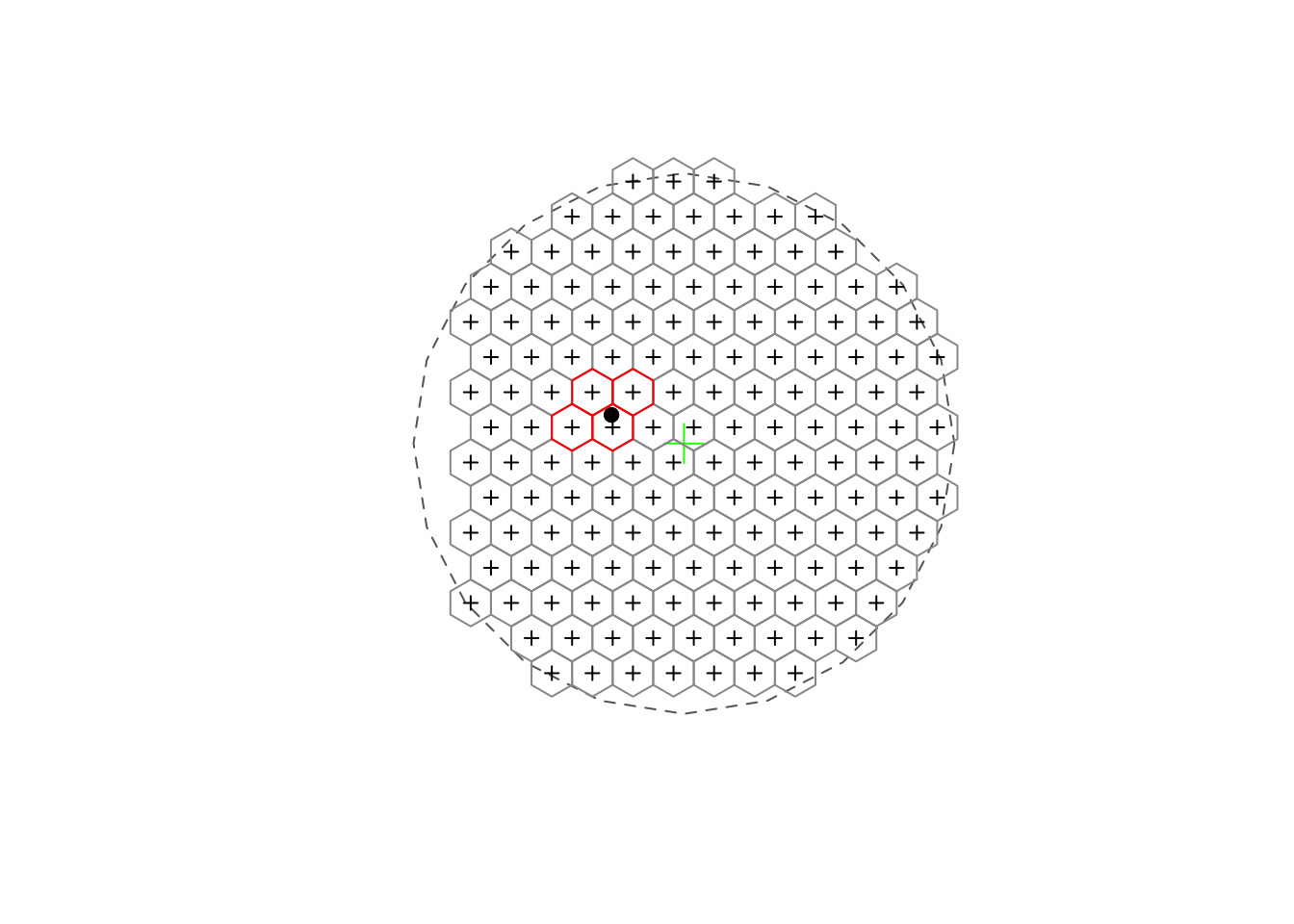

Supplement: Supplementary file 2 — Fig S2 [file ECE3-11-12307-s003.jpg]

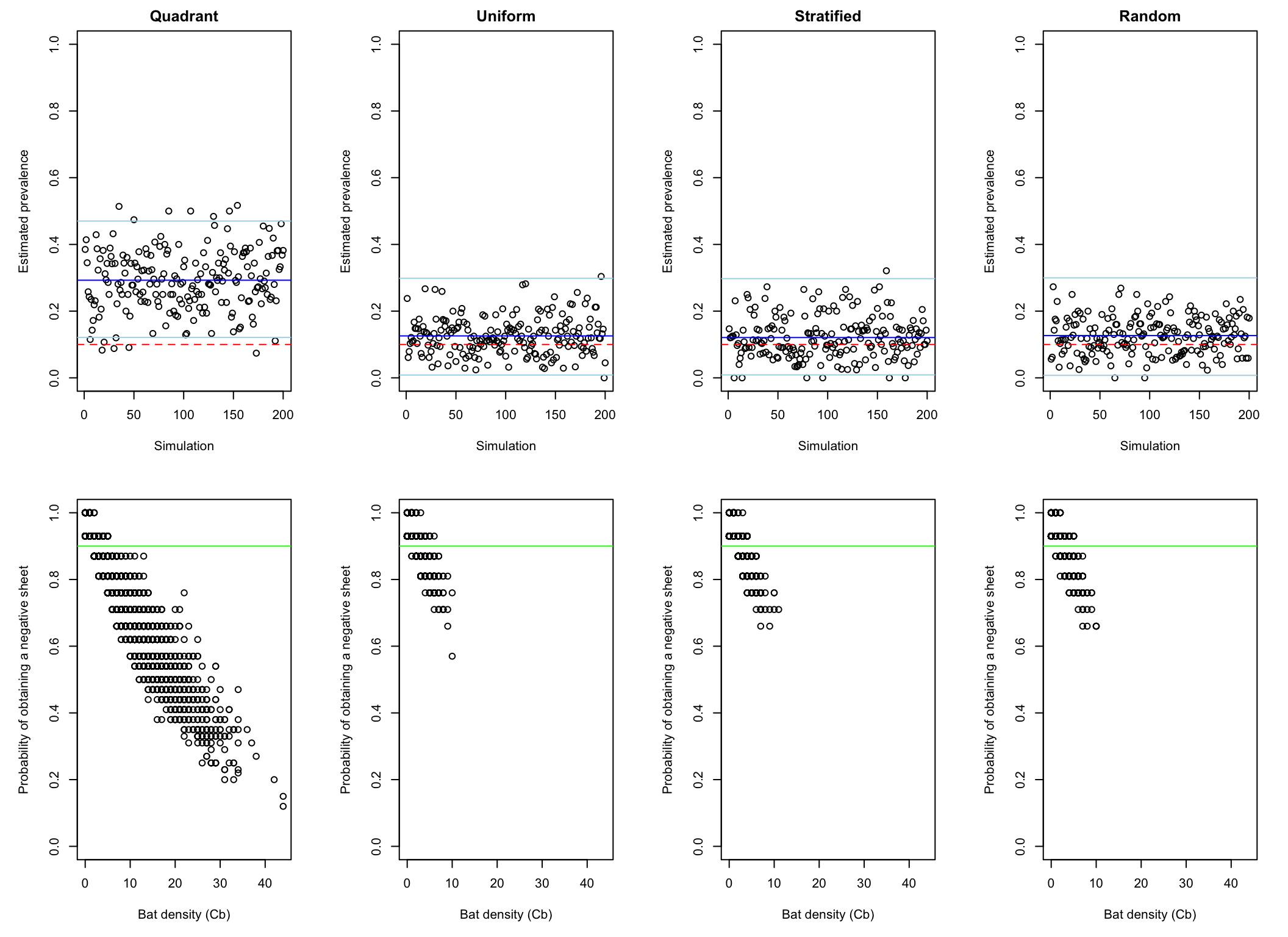

Supplement: Supplementary file 3 — Fig S3 [file ECE3-11-12307-s004.jpg]

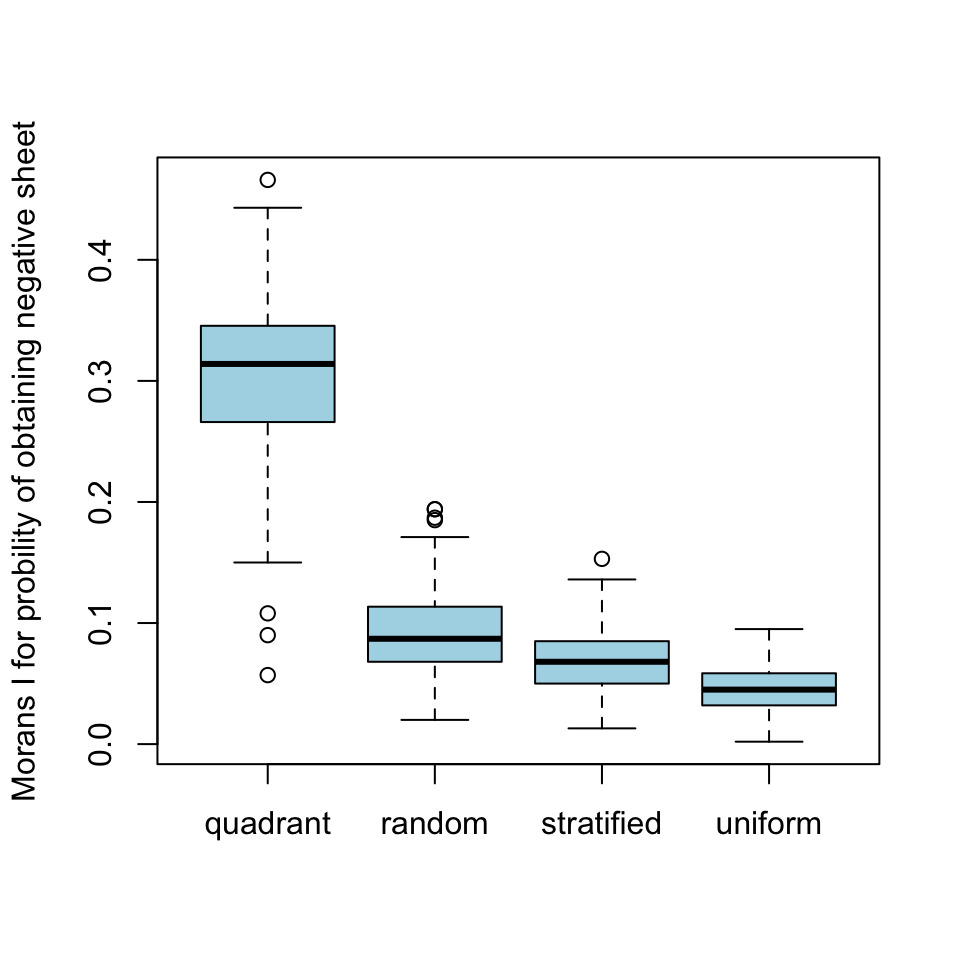

Supplement: Supplementary file 4 — Fig S4 [file ECE3-11-12307-s008.jpg]

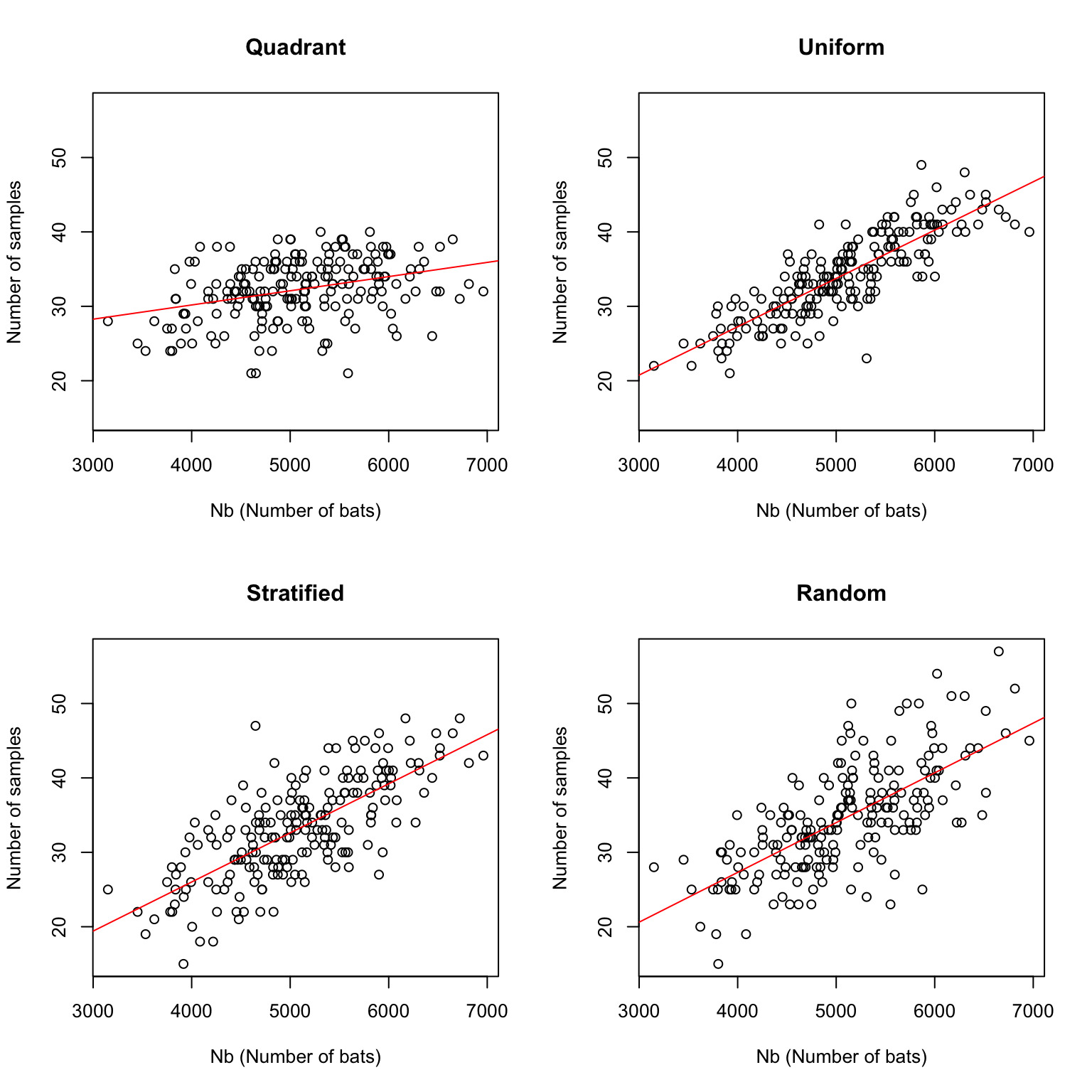

Supplement: Supplementary file 5 — Fig S5 [file ECE3-11-12307-s007.jpg]

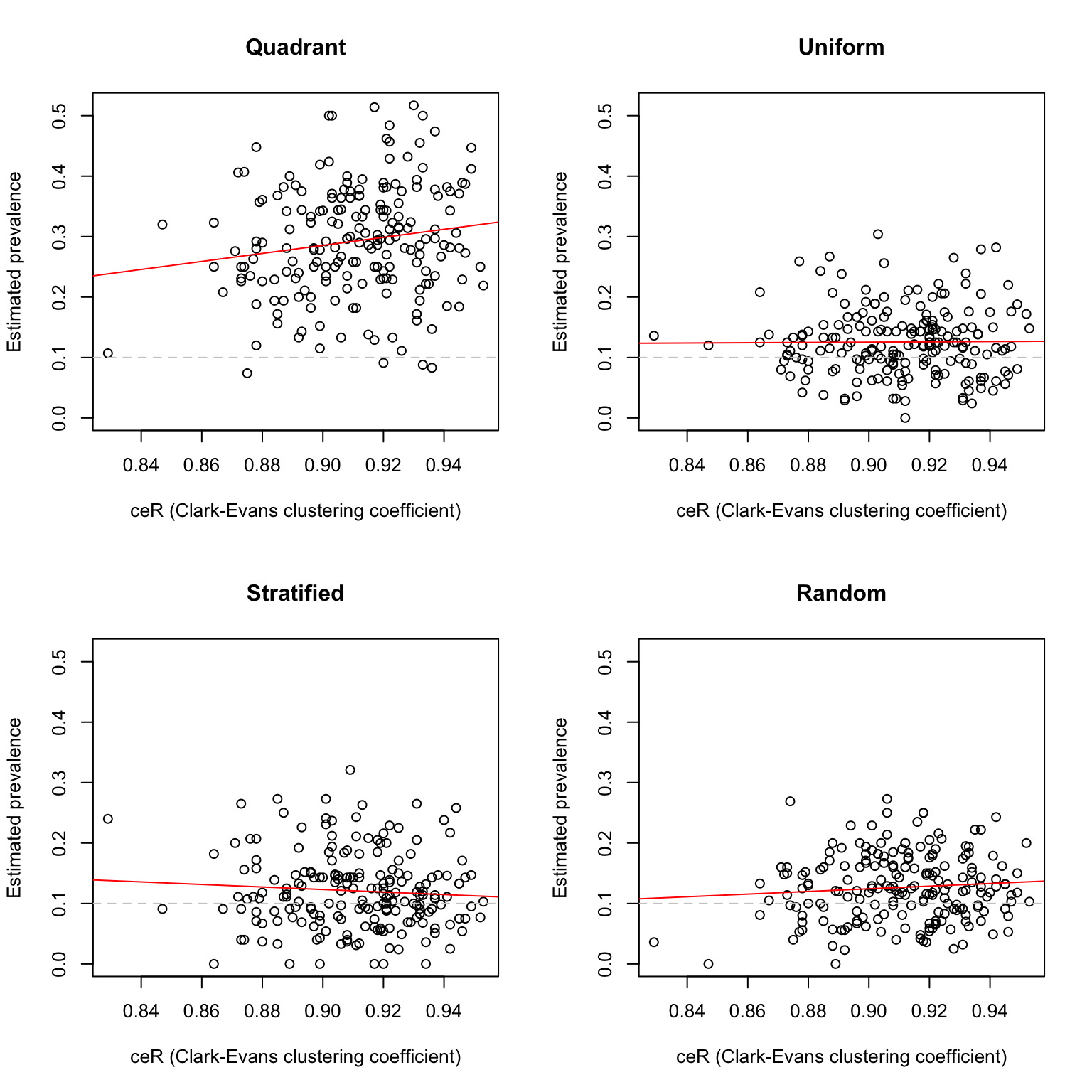

Supplement: Supplementary file 6 — Fig S6 [file ECE3-11-12307-s002.jpg]

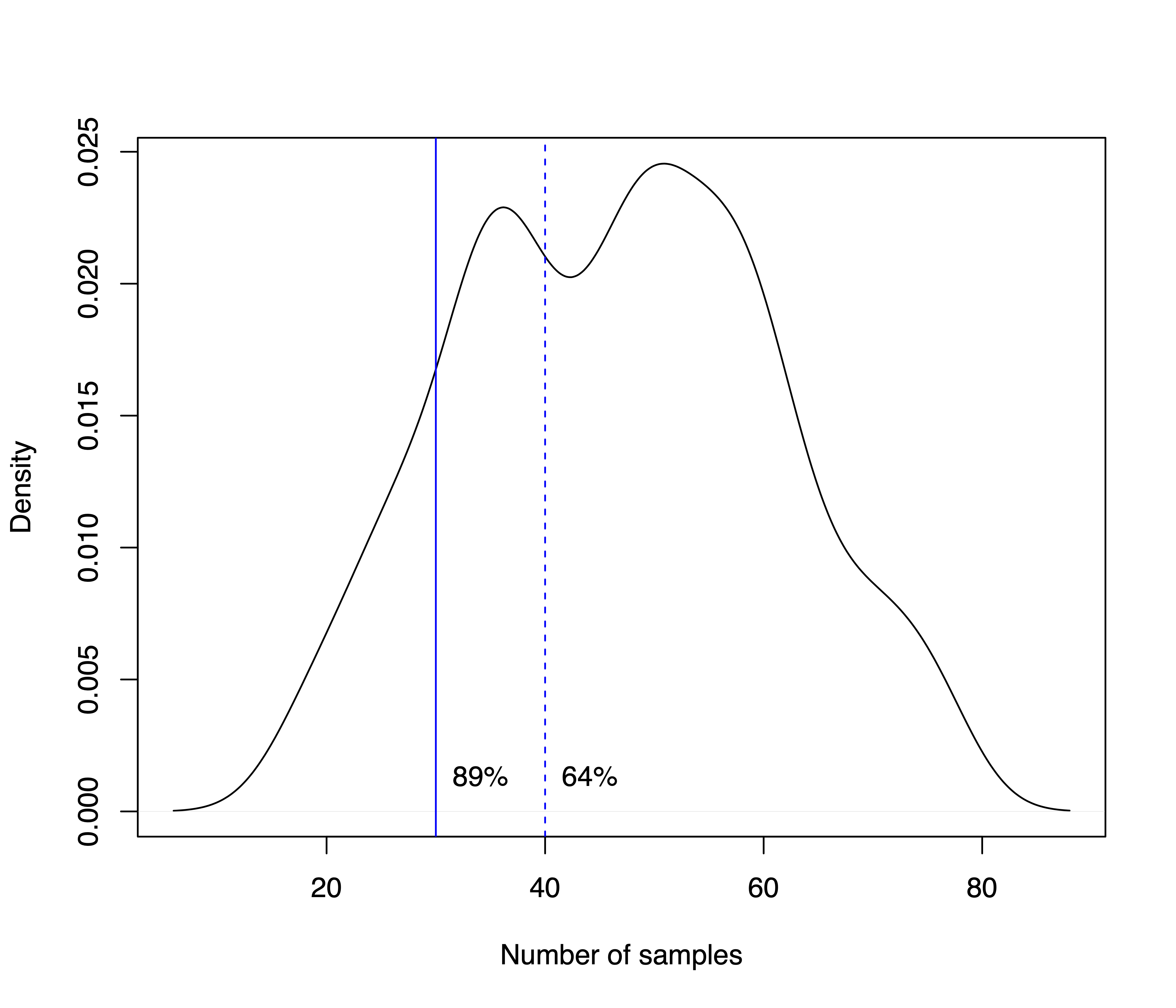

Supplement: Supplementary file 7 — Fig S7 [file ECE3-11-12307-s006.jpg]
