# Supplementary material for: Optimizing noninvasive sampling of a zoonotic bat virus
Source: Ecol Evol. 2021 Aug 27;11(18):12307–21. doi: 10.1002/ece3.7830 (PMC8462156; doi:10.1002/ece3.7830)
Supplement: Supplementary file 8 — Table S1 [file ECE3-11-12307-s001.pdf]

Table S1: Results of comparison of Generalized Additive Models (GAMs) fit to Hendra virus prevalence data. Mean bias gives the difference in the mean estimated viral prevalence predicted by the GAM using individual-level data compared to models using data that is aggregated at two levels: pooled-level samples that are aggregated within each sheet quadrant, and sheet-level samples that are aggregated within each sampling sheet. The magnitude of bias shows how many times higher the mean estimated prevalence is in models using the pooled- and sheet-level data compared to the individual-level data.

Source	Sample level	Mean bias	Magnitude of bias
Field data	Pooled	0.07 (-0.04–0.3 95% CI)	3.2 (0.31–6.5 95% CI)
	Sheet	0.21 (-0.02–0.71 95% CI)	8.5 (0.47–23.2 95% CI)
Simulation	Pooled	0.06 (-0.06–0.38 95% CI)	2.5 (0–12.1 95% CI)
	Sheet	0.21 (-0.06–0.73 95% CI)	6.9 (0–39.4 95% CI)
